# Assessing Medical Students’ Perception of Implementing Journal Club Activities: A Qualitative Study

**DOI:** 10.7759/cureus.48726

**Published:** 2023-11-13

**Authors:** Roaa Aljumaa, Reem Elmokattaf, Mohammad Aljumaa, Haifa Almuhanna, Marukh Rashid, Ismail A Abdullah, Abdul Rahman Sukar

**Affiliations:** 1 Medicine, Alfaisal University College of Medicine, Riyadh, SAU

**Keywords:** medical education, journal club impact, student’s perception, undergraduate, journal club

## Abstract

One of the most significant extracurricular activities throughout the medical students’ college years is journal club. It aids in the development of several abilities in students, including reasoning skills, searching ability, and social skills like giving and taking criticism. Our study's objective is to comprehend how students felt about the installation of journal clubs. This research was carried out at Alfaisal University College of Medicine, a private institution of higher learning in Riyadh, Saudi Arabia. Twenty-two undergraduate medical students were interviewed as part of our qualitative, interview-based approach to data collection. We made an effort to obtain a deeper understanding of how they perceived the various facets of the journal club, including the difficulties they encountered, the advantages of participating and presenting, as well as their interaction with their team and mentors. Four primary themes emerged from our thematic data analysis: (i) interaction between participants and their mentors, (ii) challenges that the participants faced during their preparation and presentation, (iii) advantages participants gained from their experience in the journal club, and (iv) participants’ experience with communication and teamwork. The findings of this study indicate that students gain many advantages from their participation; however, they still face some challenges, which provide a platform for improving journal clubs for a more beneficial experience for the students. A more targeted mentoring and improved journal club scheduling that takes into consideration the students’ schedules may help students have a more positive journal club experience.

## Introduction

Journal clubs have become an integral part of medical education and training, offering medical students a unique opportunity to enhance their skills in critical appraisal, evidence-based medicine, and professional development. These clubs regularly hold gatherings where participants discuss and analyze articles from scientific journals. Medical literature is constantly evolving, and it is crucial for medical students to be able to assess the quality and relevance of scientific studies. By presenting and discussing journal articles in a supportive and intellectually stimulating environment, students learn to critically evaluate research methodology, statistical analysis, and the validity of study results. This skill is essential for providing high-quality patient care as it enables students to make informed decisions based on the best available evidence [1-6]. Journal clubs are currently one of the most known ways to introduce students to evidence-based medicine, which is defined as "conscientious, explicit, and judicious use of the current evidence in making the best decisions about the care of individual patients" [7,8]. 

Therefore, journal clubs play a vital role in staying up to date with the latest medical research. By regularly reviewing and discussing recent publications, medical students can remain aware of the advancements and changes in their respective fields [5]. 

Considering how medical knowledge is expanding rapidly, journal clubs act as a means to bridge the gap between academic learning and clinical practice. Through journal clubs, students can identify emerging trends, new treatment modalities, and evidence-based guidelines, enabling them to provide the most current and effective care to their patients. Journal clubs also foster critical thinking and problem-solving skills essential for medical practice. By actively participating in discussions and presenting their own analysis of research articles, medical students develop the ability to think critically, question assumptions, and challenge prevailing dogmas. These skills are fundamental not only for generating new knowledge but also for identifying gaps in existing research and contributing to ongoing scientific discourse. Encouraging students to actively engage in journal clubs helps cultivate an analytical mindset that translates into more effective and innovative clinical practice [1-6]. 

Another significant impact of journal clubs on medical students is the emphasis on lifelong learning. By familiarizing themselves with the process of critically reading and appraising scientific literature, students develop habits of continuous learning and self-improvement. In a rapidly evolving field like medicine, it is important for medical professionals to stay abreast of new discoveries, changes in guidelines, and emerging technologies. Journal clubs instill in students the importance of seeking out and critically evaluating new information throughout their medical careers [1,3]. 

A study conducted in 2021 showed that a journal club can be incorporated into an undergraduate curriculum to assist students in developing their analytical skills, understanding of biostatistics, and motivation to perform future research. Most of the pupils showed receptivity to the introduction of journal clubs [9]. Participating in journal clubs helps students develop effective communication as well as professional skills, as they learn to clearly present their thoughts, engage in debates, and defend their opinions. These skills are invaluable in a healthcare setting, where effective communication is essential for successfully interacting with patients, colleagues, and other members of the healthcare team. Moreover, journal clubs provide a platform for networking and collaboration among students and faculty members. By bringing together individuals with shared interests and a passion for evidence-based medicine, journal clubs foster a sense of community, facilitate mentorship opportunities, and create a supportive learning environment [1-6]. Thus, journal clubs have a significant impact on the education and training of medical students. By improving critical appraisal skills, staying up to date with current research, fostering critical thinking and problem-solving abilities, promoting lifelong learning, and contributing to professional development, journal clubs play a vital role in preparing medical students for their future careers. These clubs create a dynamic and engaging environment that cultivates a spirit of inquiry, innovation, and evidence-based practice among medical students. The purpose of this study is to assess medical students' perception of implementing journal club activities as it includes the basic principles of adult and active learning in a traditional education tool to maximize the opportunities to expand critical thinking, advance communication skills, and establish personal confidence in these skills.

This article was submitted as an abstract to the 18th Annual Research Conference in King Fahad Medical City Hospital, which will take place on 6-7 December 2023. 

## Materials and methods

This study was conducted at Alfaisal University in Riyadh, Saudi Arabia. At Alfaisal University, journal clubs are part of the extracurricular activities conducted by one of the university's clubs; interested people sign up for the journal club and are then interviewed and selected to present based on the interview. We used a qualitative case study approach to explore medical students’ insight regarding the implementation of journal club activities at the College of Medicine, Alfaisal University. Four group interviews were conducted as part of this study in January 2023. Alfaisal University Institutional Review Board approved the study (approval number: IRB-20205). 

Interviews were conducted with multiple open-ended questions to provide us with a comprehensive picture of the journal club's impact on the students (See Appendices). Using this strategy, we were able to create a framework for creating knowledge from live experience, which we then used as the foundation for a deeper comprehension of the journal club's influence. Participants were able to interact and have discussions in focus groups, and the group dynamics offered insightful information. Collaboratively creating sense together, exchanging diverse viewpoints, and expanding on one other's ideas were among the activities of the participants. This resulted in a deeper comprehension of the subject. Furthermore, we were able to capture the attitudes and perspectives related to conducting journal club activities by bringing together a spectrum of viewpoints.

Participants in the study were both male and female medical students from Alfaisal University, from first year until internship, who had given at least one journal club presentation and were open to taking part in a focus group. First of all, the database of the research office was used to assemble a list of students who participated in the journal club previously. Then, we sent an email to all students, explaining the research objectives and what the interview entails, requesting them to take part in the study. We received replies from 22 students who we then divided into four groups, each group including four to eight students. A lead investigator (RE) with previous experience in conducting qualitative interviews led a total of four one-hour interviews in English. Of the 22 medical students interviewed, two were first-year students, nine were second-year students, nine were third-year students, and two were fourth-year students. We conducted interviews up till the point of thematic saturation where the same themes came up again, and the data did not reveal any brand-new or significantly distinct topics.

We developed an interview guide with semi-structured, open-ended questions so that participants could express various viewpoints and our team could gather comprehensive data. The interviews were recorded on two devices using the “voice memo” application on two of the co-investigators' phones with consent from the participants. We did the initial interview, and then we recorded, transcribed, and examined the responses. We made a few modest adjustments to the interview guide taking into consideration the interviews that were already conducted to better communicate our meaning and gather more pertinent data. During the interviews, there was a lively exchange of ideas and spontaneous conversation that covered various facets of the topic. All of the questions were asked by the interviewers in a similar general order. All files could only be accessed by the principal investigator (PI) and co-PIs in order to protect participants’ privacy. 

An inductive thematic framework analysis was applied for the data analysis in this study, allowing us to classify conversational text and spot trends. We were able to recognize codes and find connections between thoughts and codes through repeated readings of the transcripts, and we kept coding until data saturation was reached. 

A number of descriptive and in vivo codes were created during the data analysis phase. Seventeen codes in all were found through the four interviews. The participants' best examples, the origin of the code, and its definition were all included in each code's components. Group consensus was used to settle coding disputes, and codes were compared to verify inter-coder dependability. We examined the data within these codes and then generated descriptive memos. The memos contained chosen examples from the transcripts of the interviews as well as a brief discussion of our study of these codes. The themes covered in the results section came from these memos. 

## Results

As shown in Table 1, while evaluating students' perception of journal club implementation, we came up with four major themes: (i) the interaction between participants and their mentors, (ii) the challenges that the participants faced during their preparation and presentation, (iii) the advantages students gained from their experience in the journal club, and (iv) their experience with teamwork and communication. 

**Table 1:** Findings from data analysis

Theme	Major findings
Interaction between participants and their mentors	Involved dissecting research material, explaining complex concepts, and providing feedback on presentations and papers.
	Students' experiences with mentorship vary, with some perceiving the feedback as harsh, particularly for newcomers, and a desire for more consistent support and individualized attention.
Challenges that the participants faced during their participation and preparation	Participants expressed a need for improved organization, suggesting the assignment of TAs to each paper.
	Comprehending complex research papers, particularly in terms of critical appraisal, was a common problem and more guidance was required.
	Timing and schedule conflicts were a significant concern, with participants noting clashes with other academic responsibilities
Advantages participants gained from their experience in the journal club	Overcome the fear of public speaking. And improve their presentation skills.
	Social skills enhancement and collaboration with expertise in the research field.
	Gain the ability to understand different research methods and analyze the data in distinct parts of the research.
Participant’s experience with communication and teamwork	Members with diverse academic levels boost collaborative communication and help in guiding and clearing concepts.
	Allocating roles and maintaining a harmonious group dynamic improves conflict resolution and teamwork skills.

Theme 1: interaction between participants and their mentors 

Interaction between students and their mentors is an essential aspect of participating in the journal club. Mentors played a crucial role in enhancing participant’s ability to comprehend research materials. Several participants agreed that mentors helped them dissect papers with complex language and structure and simplify them for presentation purposes. For instance, a participant stated:

Initially, I struggled with the paper; the mentor helped me out by debulking the paper and made me present it in an easy way.

This support contributed to a significant increase in participants' ability to comprehend intricate research concepts that could potentially assist them in creating their own research papers. 

Similarly, participants also stated that sometimes they didn’t know where to begin or what to do due to lack of experience and, therefore, they appreciated the exhaustive efforts put in by their mentors to guide them. As one participant said about the mentor they interacted with:

He helped us, not only me but also my group. He helped us understand some parts of the study we didn't take, or we weren't able to understand.

Participants consistently highlighted the role of their mentors in guiding them through the unpivoted path of the research process. One participant, for instance, noted:

Our mentor dissected the picture for us in the beginning, he showed us what parts he thought we must improve on, what parts we need not. And he was like there every step of the way.

This active involvement and guidance provided by mentors was acknowledged as valuable and significant, particularly for those who were new to the field of research. 

Participants expressed a wide range of outcomes from their experiences regarding the teaching strategies utilized by mentors as well as explaining challenging concepts. A participant shared:

The mentor tried to explain as much as he could to help us even though I was a first-year student, so some of the topics were new to me. The way he explained was very wonderful.

This positive perception of mentorship highlighted the mentor’s dedication to ensuring comprehension and clarity. 

On the contrary, few participants quoted that their mentors took a hands-off approach, as one participant mentioned, “In contrast, the mentor lets you do it." This approach was noted as a unique approach to mentoring, providing participants with a platform to express more autonomy in their research process. 

Despite the overall positive feedback, participants did encounter challenges in their mentorship experiences. Some participants anticipated more extensive guidance, especially for newcomers to the field. A participant mentioned:

I wish we had someone who was more available every step of the way or would meet us individually and target each component of the presentation.

Such challenges in mentorship highlight the significance of aligning a variety of mentorship styles with participants' needs and expectations. 

In addition to that, sometimes participants anticipated that the mentor would draw attention to key elements of the paper, but this expectation was not always satisfied. As mentioned by one participant:

I was expecting them to point out anything that we missed in the paper because we did not have a very elaborate approach of actually reading everything through, and I thought that, let's say, there are certain points that we did not feel were very important, that they might have been important but at our level, we don't really know, so I thought those things might be pointed out or that the doctor would say you didn't mention this specific point or this point was mentioned here and why did you move it to a different section, and so on.

On the other hand, they had expectations as stated by one of the participants, to help them with analyzing the paper at their pertinent level:

Like I said, having a good mentor and going over previous journal clubs or Googling what parts of paper you should contain, what makes a good paper, and things like that

Furthermore, unfortunately, some students also expressed their negative overall experience with their mentors due to different reasons such as time constraints. One of the participants mentioned:

We only got him a day before the actual journal club and one of my colleagues had to change an entire results section, so our experience with our mentor was not actually good.

There were many other challenges faced by presenters, which are discussed in the next section. 

Theme 2: challenges that the participants faced during their preparation and presentation 

Participants during the interviews expressed experiencing several challenges that they faced during their preparation for the journal club presentation. For example, participants underlined the need for further organization in the structure of journal clubs. A certain number of participants caused disquiet in the organization of team meetings and responsibilities. For instance, one participant mentioned:

I think they might have a little bit more organization with the meetings and the conflicts between the team. It seems that one doctor is doing everything, and then that might be the reason why he's not able to go through the paper.

Participants gave prominence to improved collocations, such as assigning teaching assistants (TAs) to each paper, which could potentially alleviate these issues. 

Furthermore, a portion of participants described a sense of disorder and messiness within the journal club process, indicating a need for a more streamlined course of action. One participant remarked:

I feel like it could have been more organized. I felt like it was very kind of cluttered and messy sometimes.

Another difficulty participants encountered was comprehending challenging research articles. Some participants had trouble understanding the material they were given. One participant shared:

When I first saw the paper, I was like, it's kind of complicated, but it was also interesting, and I didn't know how to understand it.

Participants met challenges when evaluating research publications analytically and in terms of their technical features. As one participant put it:

For my part of the critical appraisal, it was kind of hard to understand because it's not like the other parts where you just read and then you summarize it. No, it's like you have to criticize the paper and you must understand what a good research paper contains to be able to critically appraise it.

Some of the participants reflected on their experience as first-year medical students joining the journal club, and going through research: 

That’s what I'm saying. It was a bit overwhelming. I remember we had the meeting and it looked like everyone knew what was going on and I was sitting there and I was a bit clueless. 

Likewise, they also had trouble delivering the complicated components of an article in a simple fashion to the audience, as one of the interviewees argued:

I guess for me, the only challenging thing was how can we make all those paragraphs which are very vague at the beginning to very simple things and to a way that I understand so that I can explain to the audience.

Students also had difficulty grasping the research papers’ technical aspects, particularly those pertaining to surgical procedures. One participant said:

We struggled as a team a lot with the surgeries that were listed in the actual paper. So, we had difficulty understanding what's going on and what they are doing here, what are they respecting, what are they moving, and it was more of the technical bits of the paper because it was a bit advanced. 

Stage fright and presentation anxiety were not uncommon challenges outlined by participants. Some participants exhibited nervousness about presenting in front of their peers and faculty. One participant noted:

It’s about the mentality, like how you think. If you really are too paranoid, like overly extremely paranoid about all these people, all of them, they're gonna judge me. They're going to say this, they're going to say that. Oh, look at me. I made a mistake. Oh, no, no. I'm the worst person. It's all about what's in your head and what goes on in there.

Another participant shared their stage fright, saying:

I feel like it was scarier because someone who was like a doctor was going to be watching me present, and I might say stupid things, you know? Am I making sense? So that was a bit scary.

Timing and scheduling were significant troubles mentioned by participants. A portion of the participants stated that the timing of the journal club sessions clashed with their academic responsibilities, such as exams. A participant observed:

Maybe one disadvantage is that people wouldn’t show up if it was close to exam season. A small audience would be disappointing.

Others observed that tight deadlines and last-minute notifications made their experience of preparation difficult. Additionally, the journal club's timetable periodically interfered with participants' academic plans, which addressed stress and complicated time management to their concerns. One participant spoke about having to prepare for both an exam and a journal club presentation, saying:

I remember it very well. My journal club was on Thursday, and I got accepted on Monday. I had one week, six days actually, for an exam. And my journal club was in three days. So now I must do something, and I didn't know how I managed to manage my time. Divide the day between preparing for a journal club and studying for the exam.

Even though this was the experience of most of the participants, some of them had a different experience regarding the timing of the journal club. One of the students said:

My experience was the opposite, we met up early in the week and we had a lot of rehearsals, and we practiced a bunch of times.

Similarly, another student noted:

Our journal club was perfect in terms of the timing because we prepared in the break and then we presented two or two days after the university opened.

Theme 3: advantages participants gained from their experience in the journal club 

Journal clubs offer numerous advantages to the participants. One of these benefits is the skill of public speaking. Although students struggled with stage fright, they nonetheless thought that journal club helped them face the public in a more suitable way. Many students have stated that the journal club provided them with an opportunity to boost their self-confidence and public speaking skills, for instance, a student mentioned:

Overcome stage fear and now I am dealing with research paper, which is new for us as medical students.

Another student elaborated:

It gave me a lot of exposure to the public. Public speaking, which I wasn't comfortable with until I joined my journal club, significantly boosted my confidence.

Additionally, it improved their presentation skills; one of the students shared:

Presentation skills are crucial because you need to know exactly what to include in the slides, how to make it concise, and how to ensure the audience understands your message.

Consequently, these presentations enhanced the students’ ability to communicate with the audience and significantly increased their confidence. Some students even suggested some ways to help with the fear of public speaking:

Um, honestly, I'd say practice will make it better. But the only way to actually combat this is to go for it. If you can't, you can't read books or read manuals on how to present or like talk on stage. You're just gonna have to do to finally get rid of it.

Another advantage that some students pointed out is that their teamwork and social skills developed due to the journal club, as effective collaboration with team members was necessary for smooth presentations. One student highlighted the advantages as follows:

I would categorize them into three areas: presentation skills, teamwork, and research understanding. You learn how to interpret research and read it, which undoubtedly helps when explaining it to future researchers.

Interestingly, towards the end of the journal club, many students mentioned gaining research understanding abilities through participation in the journal club as one of the benefits they gained from participating. This allowed them to comprehend research terminology, such as research methodology, and to interpret research results as one participant said:

You learn a lot of soft skills in terms of how you do research; for example, other articles related to the article or how to use research platforms. Also, you have these benefits of connecting with other people and gaining more confidence.

Another student mentioned:

It teaches you how to analyze and interpret the results of your research and how to critique it.

Another interviewee agreed:

Yes it helps you with the terminology and the language of the research paper so you could understand any paper that, uh, you read in the future.

One of the participants elaborated on another skill that people gain aside from the research experience itself, and that is the acceptance of criticism:

Other than actually reading the research paper and getting all the knowledge from that article. The fact that you're explaining it to someone, and let's just say they do understand it, that sort of quote-unquote validation that you get crazy. And another thing is, how it kind of helps you as a human being to actually accept criticism yourself. And it really helps with building character, I guess.

Moreover, some students commented on the fact that since journal clubs put time pressure as they’re combined with the students’ main courses, it helps participants attempt different ways to try to manage their time. Journal clubs also helped them manage stress and anxiety, as they had to balance journal club commitments with their studies and exams. Another student added:

For this, I think it teaches you how to manage your time effectively despite the anxiety and pressure they put on you. So, yes, it helps with future management.

Furthermore, participants agreed that journal clubs also aided in their academic performance, particularly in a biostatistics course, Basics of Biostatistics and Epidemiology (BEP), that students are required to take in their medical school. One participant noted:

I think someone mentioned the, what's it called? BEP? I think it would help with that. Honestly, I, I didn't know anything about like p-value or legends or significance, but then, But I, I worked around it, you know, I tried to like do my own research and try and find out what each of those things are. And I feel like, but just like a little bit of background knowledge. I think I'll have like a slight upper hand than, you know, anyone taking BEP with me. Yeah. When that happens.

Another participant elaborated on how it could help with this particular course by giving an example:

I would say that, if you read a paper, it'll simplify some courses in the second year. Like BEP uh, we had, one first year in our group and he had the result part, uh, even though he, he has a traumatic event, but I believe if he got a BEP he will understand, uh, most of the things. So, applying knowledge, I would say.

One student elaborated on the fact that journal clubs can help students with searching for information as it is a needed skill for students. The participant said:

Academically, it'll help them know how to read. Because this is a very important thing and how to search. Because when I come across a word that I don't know, some people just translate, some people will look for it in other articles, in other contexts. So, all of this, like the ways to search for something, it'll help you. Now when I see something I don't understand the way, I think I need to face this and now understand that this helps me academically. Cause now in the lecture, if I see something I don't understand. Now I have the skills of searching and knowing how to counter these things. Like the unknowns for me.

Finally, another significant advantage of the journal club is that it could be an excellent platform to meet people who would help students interested in research. One student stated:

It's beneficial because you get an opportunity to speak with the moderator afterward and meet like-minded people focused on research, so potentially maybe in the future if you want a research paper that requires more people, you could invite them to participate with you.

Theme 4: participants’ experience with communication and teamwork 

Many students noticed that the journal club consisted of multiple students with different academic levels, which was a unique advantage. Working with students from various years allowed them to collaborate in a positively dynamic way and benefit from each other’s experiences. For example, as one student mentioned:

Being the oldest in my team, I took the lead in mentoring, but the first-year student pointed out things I hadn't thought about. It was a give and take, and we learned from each other's perspectives.

These varying academic levels sometimes led to challenges in understanding complex research parts, especially for first-year students. However, these challenges proved to be opportunities for the team to communicate effectively and overcome any obstacles in their way. As another student said:

The paper was hard to understand, so we tried to explain it to each other, which was very helpful.

In addition, many students emphasized the importance of dividing their work equally among team members. This equitable distribution played a key role in ensuring the work flowed smoothly and contributed to a perfect presentation to the audience. One student described their experience:

We worked well together, with each of us having our strengths and roles. We divided the work equally, and it made our presentation slides smooth.

Furthermore, collaborative and accommodative communication, along with consideration of members' flexibility, were essential for the workflow. By understanding each member’s flexibility and availability, the teams ensured they didn't face any obstacles in their preparations. As one student pointed out:

Flexibility and communication were key. We accommodated each other's schedules, which helped us avoid last-minute issues.

Moreover, students mentioned that practicing together was instrumental in improving their presentation. It helped them understand and support each other in crafting the most effective presentation possible. Another student shared their experience:

Practicing together was a game-changer. We refined our presentation skills and clarified our understanding of the research.

These integrated responses provide real-life examples of how students of different academic levels collaborated and overcame challenges within the journal club, reinforcing the benefits of such diverse teamwork. Nevertheless, some students did face negative experiences when it came to group work with their teammates, because, with teamwork comes the responsibility of cooperation:

The major issue was some members were not very cooperative, so during the meetings everything would be flowing smoothly and then they just find a problem and ruin everyone's work so we fought so many times during the meetings; it was, it is, not a good experience for a short time.

Despite this experience, the team attempted to refine their behaviors, as the participant continued:

...but then we asked the team itself to interfere to solve the issue because they were really, we were struggling and then after solving the issue, everything went very smoothly and everything.

Nonetheless, other students claimed that their interactions with their group were wholly unfavorable:

I had one, he, he was like playing basketball before the meeting and we had to cancel the meeting. He came late to the journal club.

Another participant also said:

I didn’t have a good experience; some were good, and some weren’t cooperating and doing work literally last second. As I was going to present with them and not on my own, that puts extra stress on me.

There was also trouble with adequate preparation from some team members:

Members prepared last minute and because of lack of coherence between what each presenter said, some information got repeated and it was a problem in front of the doctor.

Despite all the challenges, students still attempted to overcome the different challenges in various ways. Students who had negative experiences with their mentors tried to seek help from each other, search for helpful information, and circulate it in order to have the best outcomes during their presentations. They believed that such a hindrance could also be overcome by assigning a TA to help the students. As most of the students had difficulty with the timing, it was suggested to try to wake up early, work hard, and allocate time effectively between studying and getting ready for the journal club. When it came to the challenging research articles and the critical appraisal part they had to complete for their journal club presentation, a few first- and second-year students shared their struggles. Students proposed that seniors with strong research experience or those who have already presented can assist in overcoming this. Additionally, effective TAs are crucial in supporting and demystifying the article for students.

## Discussion

The field of medicine is always changing, and journal clubs assist students in keeping up with the most recent changes and advances in their profession. 

Medical journal clubs are important for students for several reasons; they encourage critical thinking, research literacy, and a deeper understanding of current medical literature. This significance is highlighted by how most training programs include a journal club, which is frequently the framework used to teach clinical epidemiology and biostatistics [10]. Journal clubs serve a crucial role in improving students' abilities, comprehension, and preparedness for clinical practice. They give students the chance to critically assess and discuss recent medical research. In addition to that, students' communication skills are enhanced when they present and discuss research papers in a group environment where they gain the ability to clearly express complicated ideas and participate in problem-solving where they agree or disagree with certain points and ideas and express their thoughts regarding it. Therefore, journal clubs help facilitate making informed clinical decisions as they help in the development of skills required for understanding evidence-based medicine. Training in critical assessment abilities is one of the requirements for a fruitful evidence-based medicine journal club [10]. Furthermore, for students to understand the content of the articles, they must have a basic foundation of knowledge from their pre-clinical years. Students would also be able to use knowledge learned from journal articles in practical experiences in hospitals and in patient care throughout the clinical years [11]. 

The members of journal clubs benefit greatly from its execution. Some of the students believed that the journal club had benefited their academic performance. The participants in the current study emphasized how the journal club sessions helped them get ready for other presentations, such as "English and Islamic" as well. In addition, it helped them in research-related courses like the evidence-based medicine course offered at their university, by helping them lay a foundation for research and understand the terminology that would later be used in the course. The results of our study were supported by an earlier study that evaluated the effects of a journal club elective course on student learning measures in which 197 students were included [12]. Students in this study were divided into two groups: those who completed a journal club elective (n=73) and those who did not (n=124). The scores on the Pharmacy Curriculum Outcomes Assessment (PCOA) and the overall course grades for different courses were analyzed. The study demonstrated that enrollment in a journal club course was linked to better PCOA-scaled scores, both generally and specifically in relation to clinical science and literature review. Completing a journal club elective was also linked to better student performance in multiple classes. 

Journal clubs assist students in developing the abilities necessary to assess, understand, and critically evaluate the results of research, according to one of the students. Many of the participants of the current study remarked on how participating in the journal club improved their comprehension of research. They could now understand the various parts of the article, including the methodology, results, and the various research terminologies used. Similar findings were found in a cross-sectional study that was conducted among undergraduate medical students at the College of Medicine and Health Sciences, the National University of Science and Technology, Sohar, Sultanate of Oman, in February 2019 [9]. The study assessed the knowledge, attitude, and perceptions regarding the implementation of a journal club. When participants were asked whether these workshops helped them understand the primary articles they were reading, 97% of them agreed. In another study published in 2012 by one of the researchers who developed a workshop to help students read primary literature, it was evident that when the participants first viewed the papers (shortly before the workshop), 43% indicated they were worried they wouldn't comprehend them thoroughly to deliver them, and 11% said they were worried they wouldn't understand them at all [13]. 

Along the same lines, a prior study in the United States evaluated students' perceptions of a journal club organized to improve their EBP skills, including their overall satisfaction, knowledge base skills, and presentation skills [14]. The journal club was used as a component of the curriculum for the graduate-level occupational therapy course EBP. In this course, 31 students were enrolled. At the beginning of the semester, 30 participants received information about the assignment and the outcomes evaluation, and they gave their informed consent to take part in the eight-week EBP course. Students participated in two surveys (pre/post) during the course of the eight-week course to gauge their perceptions of how the journal club affected their learning. The surveys contained structured Likert-style survey items as well as open-ended ones. The responses to numerous questions were assessed by the students on a scale of 1-5 or 1-10, with 1 representing the least/lowest reaction and 5 or 10 representing the highest. Prior to the journal club activity, 18 (62.1%) respondents said that the largest obstacle to reading research articles was "understanding the statistics”. Students' comprehension of statistics was enhanced after this course activity, where 10 (38.5%) respondents cited this as the main obstacle. The guided journal club activity was well received by the students in general. On the final survey, students noted that this assignment was the one that was most helpful for learning the EBP process (average score 2.15 out of 5). After journal club, the score of people who reported that they felt at ease when contributing to the group discussions went from 3.48 to 4.04 (an increase of 16.1%). Likewise, those who reported feeling comfortable reading scholarly articles and comprehensively understanding the research article went from 3.45 to 4 (15.9% increase) and 3.14 to 3.62 (15.3% increase), respectively. After completing this assignment, students were more confident in their ability to understand research findings (from 4.48 to 5.38, a 20.1% increase). The guided journal club activity was well received and perceived by the students generally [14]. 

While journal clubs have many advantages and benefits to participants, there are still some challenges that should be addressed. To begin with, one of our participants pointed out the fact that they didn’t feel like they had the basic knowledge needed for the journal club and that they often weren’t sure which parts were important to include in their presentation and which parts weren’t. Similarly, a participant stated that they participated in a journal club during their first year of medical school and remarked on how overwhelming it was. A critical analysis of the literature on the planning, topic selection, execution, and technological integration of journal clubs in residency education was conducted in a study [15]. This was the first in a series of assessments of evidence-based best practices from the Best Practices Subcommittee of the Council of Emergency Medicine Residency Directors (CORD). The study further suggested that having a foundational level of clinical knowledge about a subject might be beneficial since it saves the learners' time to concentrate on skill development rather than learning about the topic. Another study conducted in 2016 outlines a novel approach to preparing for and delivering journal club that is built on three pillars: dialogical learning through group discussion, mentoring residents serving as peer teachers, and incorporating journal club into a set curriculum to teach students evidence-based medicine [16]. According to the study, less-experienced students struggled more with critical evaluation since they were more concerned with understanding the material than with critical analysis. Comparably, Alfaisal participants in the current study had trouble understanding complicated research papers without having the foundational knowledge needed in research. 

The participation of the students is yet another barrier to the implementation of journal clubs. The majority opposed including journal clubs in the curriculum since they take a lot of time to prepare for [17]. Due to some members' lack of cooperation, students had to deal with several problems and conflicts. It was through these difficulties and challenges that they learned how to resolve conflicts and function as a team. Having an interested person or small group devoted to the club's organization, conduct, and evaluation may be the most crucial stage in creating a successful journal club. A survey of journal clubs in United States family practice residencies found a strong correlation between a journal club's effectiveness and its single leader [17]. Although having a faculty member as the leader is crucial, other studies have shown that the longevity and effectiveness of the club are also related to the participants' active participation in planning and running the club [18-20]. Since the papers might occasionally be difficult to understand, teammates explained them to one another, which was very helpful for the cohort at Alfaisal University in the current study. The team's most experienced member took the initiative and transformed into the mentor by taking the lead and explaining how to present to the teammates. Additionally, students felt that practicing with a partner helped them deliver a stronger presentation. “We refined our presentation skills and clarified our understanding of the research.” The team approach accomplishes two things: it reduces the number of presentations to half (an important time issue), and it gives students who are anxious about class presentations some moral support [21]. One of the participants stated regarding successful collaboration: "We worked as one hand. We didn’t face any obstacles. As a team, we distributed the work thoroughly, equally and everything went smoothly."

The fact that the participants had issues with the journal club's schedule was another significant issue that came up throughout the interviews. Some of the respondents said that the journal club meetings interfered with their academic responsibilities such as exams and studying and that, in some cases, they received very short notice before the journal club meeting time. An editorial published in 2019 had the goal of underlining the value of journal club as a traditional educational instrument that combines fundamental adult learning concepts to maximize opportunities for the development of critical thinking, communication skills, active reflection, and individual confidence in these abilities [17]. The paper discussed implementing journal clubs in the undergraduate medical curriculum, and it mentioned that students may feel overwhelmed and lack excitement since they have so many courses to learn. 

Undoubtedly, for a successful journal club, mentors are an essential pillar. Supportive mentoring and direction from a devoted faculty member ensure the maintenance of the structure and systematic presentation, which aids in being innovative and creative in the presentations [22]. Support and mentoring provided by experienced faculty members in the form of guidance and technical assistance is a crucial factor [15]. Similarly, for the Alfaisal University students in the present study, sometimes the results were very challenging to comprehend, which is when the mentor's assistance was useful. In addition to that, the mentor also simplified complicated research and offered input during practice sessions considering it has a lot of graphs and figures. Similarly, faculty mentors should also train in a way to lead the discussion by ensuring that they are skilled in the critical evaluation of the selected article and organizing the interactive conversation [15]. This encouraging method guarantees the preservation of consistent patterns and systematic presentation without limiting participants' creativity. In the current study, participants acknowledged that they sometimes lacked experience and didn't know where to start, and the mentors made extensive efforts to help them. In a quasi-experimental one-group study in Mexico, it was evident that the drawbacks, however, included a lack of sufficient faculty to regulate and sometimes the faculties were already overburdened with work, so there was a lack of time for mentoring [23]. A participant in the current study similarly stated, "I wish we had someone that was more available every step of the way or would meet us individually and target each component of the presentation." 

This study has several limitations. First, because the study only included undergraduate students, we were unable to analyze the viewpoint of postgraduates, such as residents, on their involvement in journal clubs. Additionally, because the majority of the study participants were in their pre-clinical years, it was difficult to determine whether students' interest in participating in journal clubs changed over time or whether their understanding of the articles used in journal clubs improved as they advanced through medical school. Finally, this was a single-institute study. We would have gained a deeper understanding of how various students view the implementation of journal clubs had more students from different institutes been interviewed. 

## Conclusions

One of the most significant extracurricular activities in medical school is journal club since it helps students develop a variety of abilities such as public speaking, teamwork, and leadership as well as communication. Presenting in a journal club can also help with research-related benefits including comprehending research-related jargon and demystifying complex information found in publications. The goal of this study was to gain a deeper understanding of students' perspectives on their experiences with and perceptions of the implementation of journal clubs, which is the first of its kind in Saudi Arabia. The main themes in our study were the interaction between participants and their mentors, challenges faced by the participants in their presentation and preparation, advantages gained from their experience in the journal club, and the participant’s experience with teamwork and communication.

During the course of this study, it became clear that journal clubs are a valuable resource for medical students. Nonetheless, there are identifiable shortcomings in how these clubs are organized, and we can explore ways to address these issues for the betterment of students' engagement in journal clubs. Some of the potential solutions, as suggested by the students, include having a TA. Given the significance of journal clubs, additional research in this area is necessary. By covering both pre- and post-graduates and different establishments, a broader understanding of how journal clubs are perceived can be obtained. This contributes to improving journal clubs so that more individuals would join and gain from them. 

## Figures and Tables

**Figure 1:**
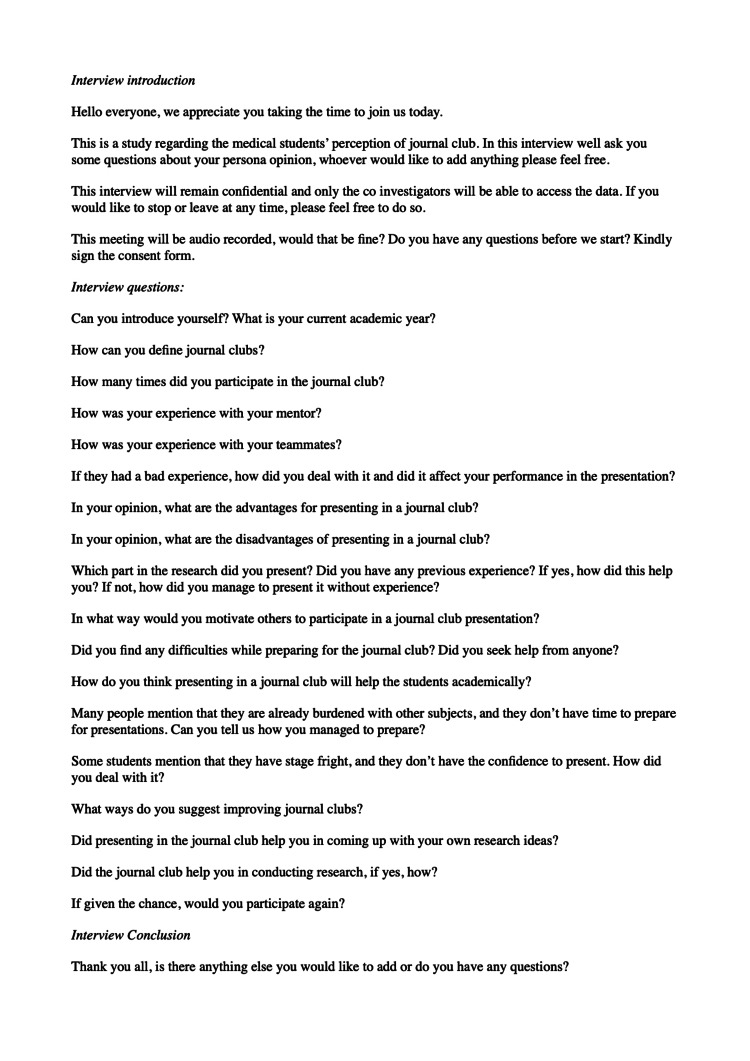
Study Instructions and Questionnaire The interview was semi-structured with open-ended questions; therefore, there were main questions asked throughout all interviews. In order to conduct this research, all interviewers adhered to the instructions listed. However, a few questions were added in different interviews that came along with the flow of the discussion of the participants.
